# Therapeutic Effect of His-Purkinje System Pacing Proportion on Persistent Atrial Fibrillation Patients With Heart Failure

**DOI:** 10.3389/fcvm.2022.829733

**Published:** 2022-02-24

**Authors:** Fei Tong, Zhijun Sun

**Affiliations:** Department of Cardiology, Shengjing Hospital, China Medical University, Shenyang, China

**Keywords:** persistent atrial fibrillation, heart failure, His-Purkinje system pacing, pacing proportion, prognosis

## Abstract

**Background:**

His-Purkinje system pacing (HPSP) combined with atrioventricular node ablation is an effective therapy for atrial fibrillation (AF) patients with heart failure (HF). However, atrioventricular node ablation has some limitations and disadvantages. HPSP combined with β -blockers reduces intrinsic heart rate and increases pacing proportion, which may be an alternative to HPSP combined with atrioventricular node ablation. This study was to assess the therapeutic effect of different HPSP proportion on AF patients with HF.

**Methods:**

The study enrolled 30 consecutive persistent AF patients with HF who underwent HPSP. Heart rate was controlled by medical therapy. NYHA class, NT-proBNP, echocardiographic parameters were assessed at follow-up. MACE was defined as the composite endpoint of readmission for HF and cardiac mortality.

**Results:**

The AUC of pacing proportion for predicting MACE was 0.830 (SE = 0.140, 95%CI:0.649–0.941, *p* = 0.018), the optimal cut-off point of pacing proportion to predict MACE by ROC analysis was 71% (sensitivity:83.3%, specificity: 91.7%). In high pacing proportion group (>71%), there were significant improvements of NYHA class, NT-proBNP, LVEF and LVEDD from the baseline in wide QRS complex (QRSd>120 ms) patients and HFrEF patients at half year follow-up, and there were significant improvements in NYHA class, NT-proBNP from baseline in narrow QRS complex (QRSd ≤ 120 ms) patients and HFpEF patients at half year follow-up, moderate but no significant improvements of LVEF and LVEDD were observed in these patients. In low pacing proportion group (≤ 71%), there were no significant improvements of NT-proBNP, LVEDD or LVEF regardless of baseline QRS duration or LVEF (*p* > 0.05).

**Conclusion:**

High pacing proportion (>71%) of HPSP can improve clinical outcomes and echocardiographic parameters in persistent AF patients with wide QRS complex or HFrEF, and clinical outcomes in persistent AF patients with narrow QRS complex or HFpEF. High pacing proportion of HPSP has a beneficial effect on the prognosis of persistent AF patients with HF.

## Introduction

Atrial fibrillation (AF) is the most common arrhythmia in patients with heart failure (HF) (1, 2). AF and HF have similar risks and mechanisms (3) related to physiological processes that initiate and sustain each other (4). Current methods to control heart rate and rhythm in patients with AF include drug therapy, radiofrequency ablation (RFA) and cryoablation, but drug therapy is sometimes ineffective and may be accompanied by adverse reactions. Catheter ablation has a high recurrence rate of AF (5), especially the recurrence rate of persistent AF can reach up to 50% (6, 7). AF duration for more than 2 years and HF are identified as predictor for AF recurrence (6, 8). Therefore, the clinical treatment of AF with long duration and HF is still challenging.

2021 European Society of Cardiology (ESC) Guidelines on cardiac pacing and cardiac resynchronization therapy (CRT) proposed that CRT should be considered as a strategy for permanent AF patients with HF with left ventricular ejection fraction (LVEF) ≤ 35% and QRS≥130 ms (9), as for patients with LVEF>35% or QRS <130 ms not regarded as candidates for CRT. His-Purkinje system pacing (HPSP) including His bundle pacing (HBP) and left bundle branch pacing (LBBP) can restore physiologic activation of the ventricles and maintain ventricular synchrony *via* intrinsic conduction pathway (10). Arnold et al. indicated slowly conducted AF, CRT in patients with HF and bundle branch block (BBB) as potential indication for HPSP through assessing recent evidence and current practice (10). In 2000, Deshmukh et al. first performed HBP and atrioventricular node (AVN) ablation in patients with AF, dilated cardiomyopathy and HF with reduced ejection fraction (HFrEF), and improvement of left ventricle dimensions and cardiac function were observed (11). In 2017, Huang et al. implemented HBP and AVN ablation in AF patients complicated with HFrEF or HF with preserved ejection fraction (HFpEF), and observed improvement in symptoms and echocardiographic parameters (12). However, after AVN ablation, HBP threshold increased by 0.5–1.5V (13). AVN ablation artificially causes complete atrioventricular block and pacer-dependence, and physiology of HPSP is also different from intrinsic conduction system. Therefore, clinically physicians can prescribe β-blockers for patients with persistent AF and HF to inhibit AVN conduction function and reduce intrinsic heart rate, so as to achieve a high proportion of HPSP and the purpose of rate and rhythm control. However, there are few studies on this therapy. This study aimed to assess the therapeutic effect of different HPSP proportion on persistent AF patients with HF.

## Methods

### Study Patients

Consecutive patients who met the inclusion criteria were enrolled between October 2017 and July 2020. The inclusion criteria were the following: (1) Persistent AF with bradycardia or long RR interval, or AF recurrence after RFA, or unsuitable for RFA; (2) HF in New York Heart Association (NYHA) class was referred to II-IV class; (3) Patients were at least 18 years old and not pregnant.

Patients with any of the following conditions were excluded: (1) Severe mitral or aortic valve stenosis or regurgitation; (2) Congenital heart disease requiring cardiac surgery; (3) Severe chronic obstructive pulmonary disease; (4) Chronic kidney disease requiring long-term dialysis. The study was approved by ethics committees of Shengjing Hospital of China Medical University, and written informed consent has been obtained from all patients.

### Implantation Procedure

HBP: C315 fixed curve delivery sheath (Medtronic) was sent to the right atrium or right ventricle through guide wire *via* subclavian vein or axillary vein. The SelectSecure 3830 lead (Medtronic) was navigated into the vicinity of His bundle (HB) through delivery sheath. During the lead placement procedure, the 12-lead electrocardiogram (ECG) and electrogram (EGM) *via* pacing lead were monitored and recorded. After HB potential was identified, ECG were recorded continuously by high pressure pacing method with higher than native heart rate. Through synchronous ECG, we could determine whether HB was captured. After the ideal position was determined, the pacemaker lead was vertically screwed into interventricular septum (IVS) to maintain the stability of the sheath, and to penetrates the fibrous capsule of the His bundle. Pacing thresholds, sensed R-wave amplitudes and lead impedances were measured. The morphology of ECG at different output voltages was recorded. Thresholds of selective and non-selective HB capture were recorded. Non-selective His bundle pacing (NS-HBP) was the first choice in our center, and the acceptable threshold was ≤ 2 V/0.5 ms. If parameters of HBP were not acceptable, LBBP or ventricular backup pacing would be attempted. The lower rate for HBP was initially set at 70 bpm.

Selective His bundle pacing (S-HBP) criteria: (1) The paced QRS duration (QRSd) and morphology are both identical to intrinsic QRS complex; (2) The pacing stimulus to QRS complex onset interval (PV interval) is identical to His-QRS onset interval (HV interval); (3) The potential of HB can be determined by a narrow QRS at low output pacing and the presence of QRS broadening at high output pacing; (4) Pacing signal can be seen from the beginning of QRS complex.

**Figure 1:**
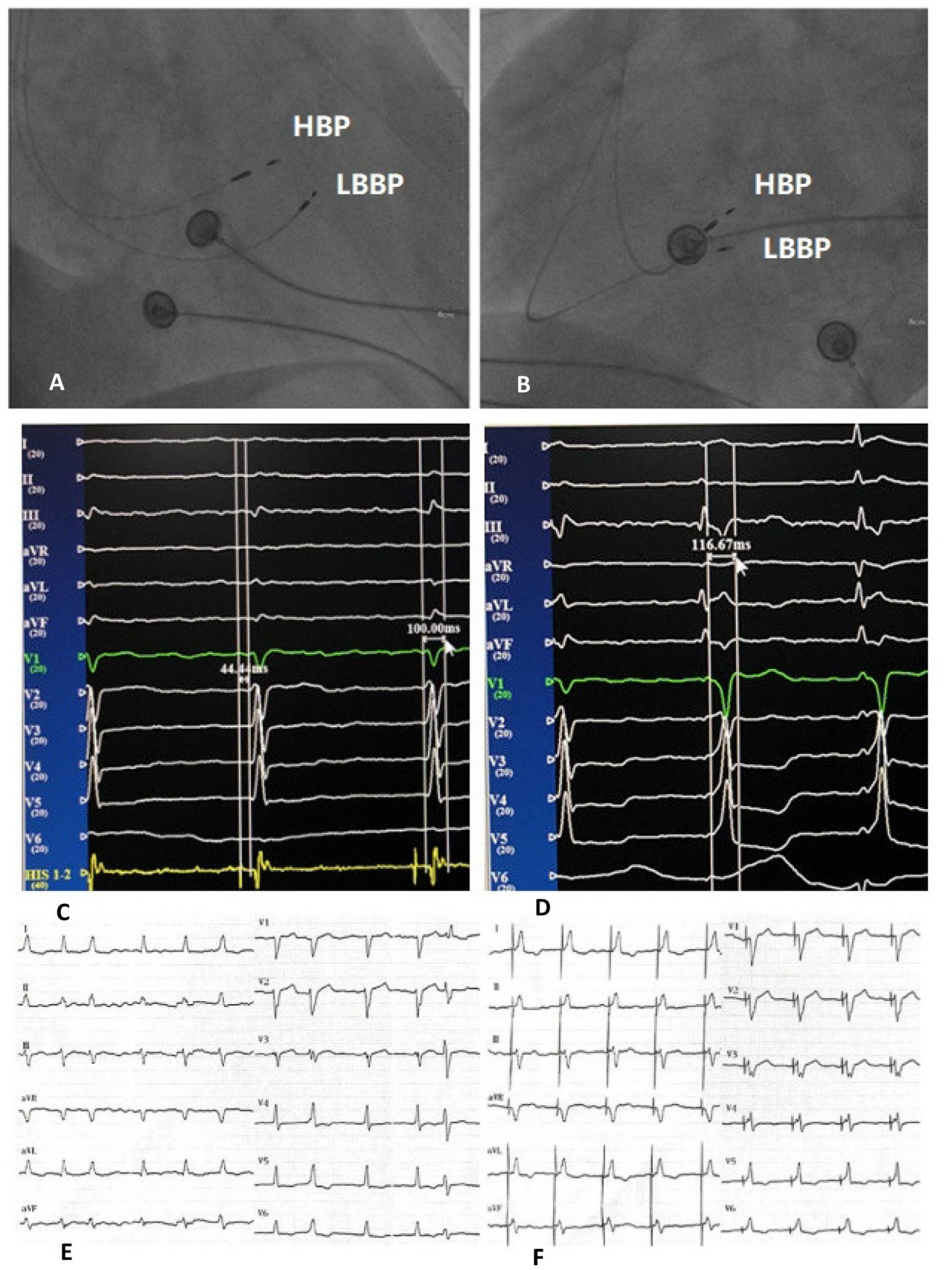
Right anterior oblique fluoroscopic projections showing location of HBP lead and LBBP lead **(A)**. Left anterior oblique fluoroscopic projections showing location of HBP lead and LBBP lead **(B)**. Twelve-lead ECG and EGM from HBP leads of intrinsic rhythm and HV interval **(C)**. Twelve-lead ECG and EGM from HBP leads of NS-HBP **(D)**. Bedside twelve-lead ECG of intrinsic rhythm **(E)**. Bedside twelve-lead ECG of NS-HBP **(F)**. HBP, His bundle pacing; LBBP, left bundle branch pacing; ECG, electrocardiogram; EGM, electrogram; HV interval, His-QRS onset interval; NS-HBP, non-selective His bundle pacing.

NS-HBP criteria: (1) PV interval is less than or equal to HV interval; (2) The potential of HB can be identified, with QRS widening at low output pacing and narrowing at capture of HB; (3) A pseudo pre-excitation wave can be immediately after HBP stimulus (Figure 1).

LBBP: SelectSecure 3,830 lead (Medtronic) was delivered through C315 fixed curve delivery sheath (Medtronic). During the lead placement procedure, the 12-lead ECG and EGM were monitored and recorded. Under fluoroscopic imaging in the right anterior oblique view, HB potential was first identified and HB region was used as an anatomical landmark. Then the sheath and the pacing lead were moved by 1–2 cm more distally along the RV septal surface toward the RV apex, and the pacing lead was perpendicularly screwed into IVS until the pacing lead helix to the left side of IVS. LBBP presented an QRS pattern of right bundle branch block (RBBB), with reduced time interval between stimulation and peak left ventricular activation time (LVAT) in leads V5 and V6. The lower rate for HBP was initially set at 70 bpm.

**Figure 2:**
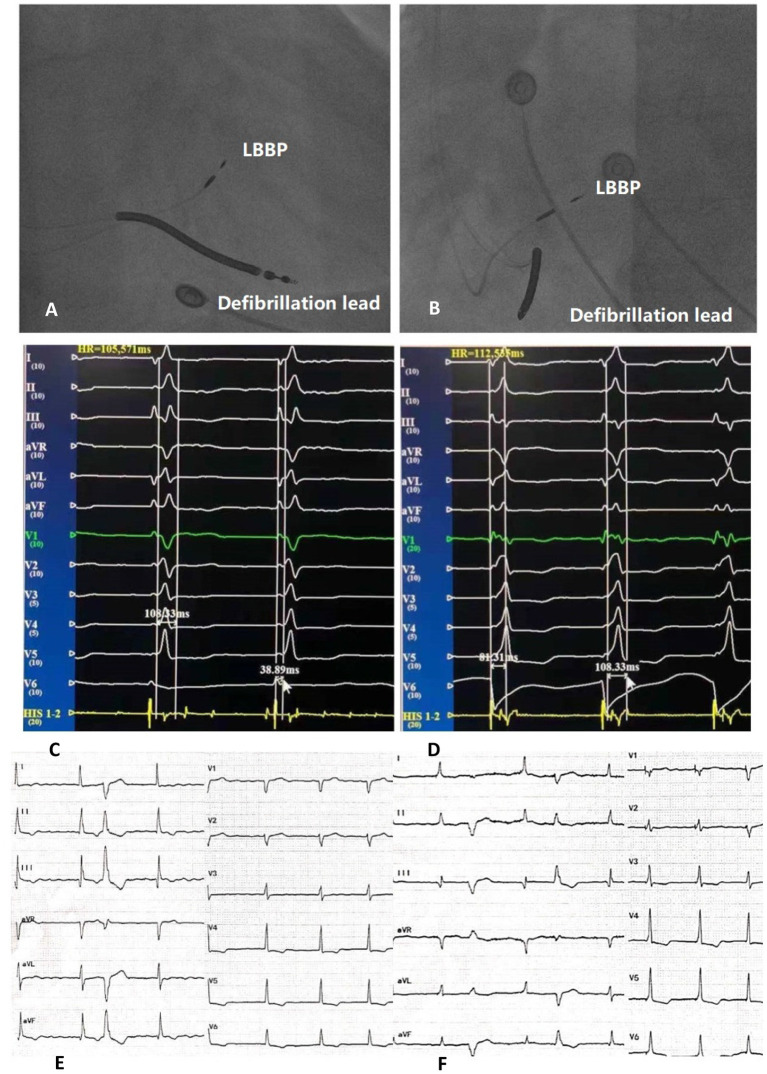
Right anterior oblique fluoroscopic projections showing location of LBBP lead and defibrillation lead **(A)**. Left anterior oblique fluoroscopic projections showing location of LBBP lead and defibrillation lead **(B)**. Twelve-lead ECG and EGM from LBBP leads of intrinsic rhythm **(C)**. Twelve-lead ECG and EGM from LBBP leads of LBBP and LVAT **(D)**. Bedside twelve-lead ECG of intrinsic rhythm **(E)**. Bedside twelve-lead ECG of LBBP **(F)**. LBBP, left bundle branch pacing; ECG, electrocardiogram; EGM, electrogram; LVAT, peak left ventricular activation time.

LBBP criteria: (1) The morphology of pacing QRS complex are RBBB; (2) Left bundle branch (LBB) potential can be identified, but LBB potential prior to V wave can not be identified during left bundle branch block (LBBB); (3) LVAT is shortened, usually <80 ms (lead V5 or V6) (Figure 2).

### Follow Up

Patients were followed in clinic at 1, 3, and 6 months. Pacing thresholds, sensed R-wave amplitudes, lead impedances and percentages of ventricular pacing were recorded at each visit. Routine ECG examination, N-terminal pro-brain natriuretic peptide (NT-proBNP) test were performed, and echocardiographic indices including left ventricular end diastolic dimension (LVEDD) and LVEF were measured during follow-up. At each follow-up visit, the dosage of β-blockers was adjusted according to pacing ratio, and the ventricular rate was controlled <60–80 beats/min as far as possible. The pacing rate was programmed to 60–80 beats/min. If necessary, the pacing rate set by the program could be increased to fulfill higher pacing ratio. After pacemaker was implanted, patients who were readmitted for HF or cardiac mortality would be recorded by phone, and the date of event would be recorded. Major adverse cardiovascular events (MACE) were defined as the composite endpoint of readmission for HF and cardiac mortality.

### Statistical Analyses

Continuous variables were expressed as mean ± standard deviation (SD) in normal distribution and median ± interquartile in non-normal distribution. Categorical variables were presented as number of patients (%). Receiver operating characteristic (ROC) analysis was performed to determine the optimal cut-off point of pacing proportion to predict MACE, and area under curve (AUC) was calculated as a measure of test accuracy. The independent sample *T* test was used for normal distribution continuous variables to compare the baseline characteristics between high pacing proportion (HPP) and low pacing proportion (LPP), Mann Whitney U test was used for non-normal distribution continuous variables, and Pearson Chi-square test was used for category variables. Paired *T* tests were performed to compare the differences between the baseline time and half year follow-up time. MACE rate curves were constructed using the Kaplan-Meier method stratified by HPP and LPP, and were compared by log rank tests. All data management and statistical analyses were carried out using the SPSS version 24.0. All statistical tests were two-tailed, and *P* < 0.05 was considered to be statistically significant.

## Results

### Implantation Results, Device Electrical Parameters and Patient Characteristics

**Figure 3:**
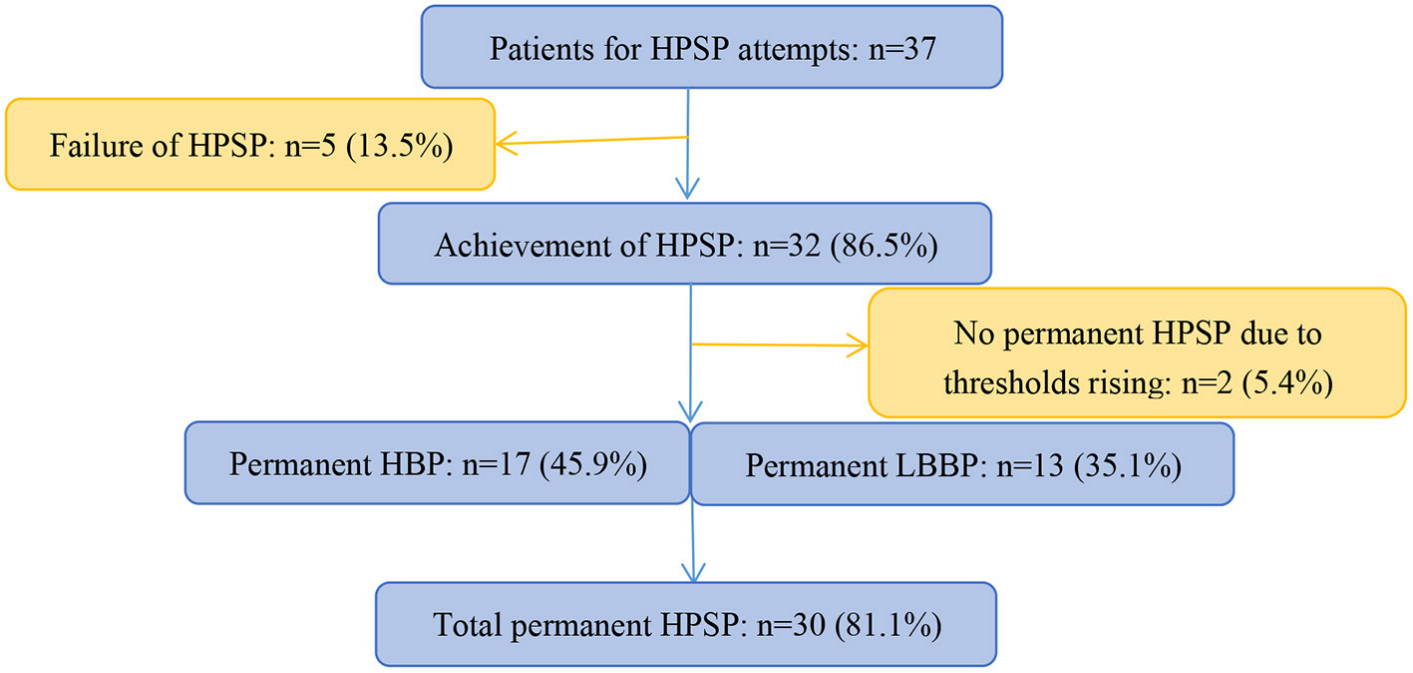
Schematic summary of study and patient flow. HPSP, His-Purkinje system pacing; HBP, His bundle pacing; LBBP, left bundle branch pacing.

In all 37 enrolled patients, HPSP were attempted (Figure 3). Failure of HPSP occurred in 5 of these patients (13.5%). HPSP was achieved in the remaining 32 patients (86.5%). Two patients did not achieve permanent HPSP due to thresholds rising (5.4%). Of the 30 patients with HPSP, 17 patients attempted permanent HBP (6 with S-HBP and 11 with NS-HBP), and 13 patients attempted LBBP. HPSP was performed in 13 patients with single-chamber pacemakers, 11 patients with dual-chamber pacemakers, 5 patients with dual-chamber implanted defibrillators, and 1 patient with CRT pacemakers.

HBP threshold, sensed R-wave amplitude, lead impedance at time of implant was 1.29 ± 0.47 V, 7.14 ± 4.13 mV, 357.5 ± 25.6 Ω, respectively. HBP threshold, sensed R-wave amplitude, lead impedance at time of half year follow-up was 1.52 ± 0.82 V, 7.29 ± 4.21 mV, 362.1 ± 45.7 Ω, respectively. LBBP threshold, sensed R-wave amplitude, lead impedance at time of implant was 0.88 ± 0.227 V, 16.19 ± 4.00 mV, 608.3 ± 69.9 Ω, respectively. LBBP threshold, sensed R-wave amplitude, lead impedance at time of half year follow-up was 0.78 ± 0.28 V, 17.25 ± 3.78 mV, 574.3 ± 77.2 Ω, respectively.

LBBB was present in 10 patients. RBBB was present in 5 patients. The native QRSd of 30 patients was 121.4 ± 29.5 ms, and the pacing QRSd was shortened to 111.8 ± 15.9 ms.

**Figure 4:**
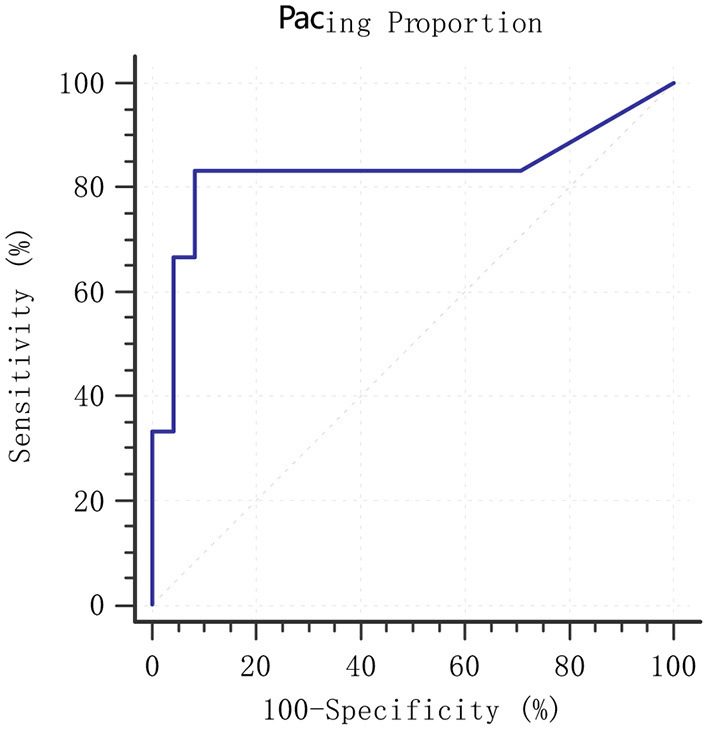
ROC analysis of pacing proportion for prediction of MACE. MACE: composite endpoint of readmission for HF and cardiac mortality; ROC, receiver operating characteristic; MACE, major adverse cardiovascular events. HF, heart failure.

The follow-up period was 15.1 ± 9.4 months. The median follow-up period was 12.0 months. During the follow-up period, 2 patients were readmitted to hospital due to HF and 4 patients died of cardiac origin. Figure 4 shows the predictive ability of pacing proportion for MACE by ROC analysis. The AUC of pacing proportion for predicting MACE was 0.830 (SE = 0.140, 95% confidence interval (CI):0.649–0.941, *p* = 0.018), indicating that pacing proportion had a significant predictive value for the prognosis of AF patients with HF. ROC analysis showed that the optimal threshold for pacing proportion to predict MACE was 71% (sensitivity:83.3%, specificity: 91.7%).

### Baseline Characteristics of Patients Under High and Low Pacing Proportion

HPP was defined as pacing proportion>71% (*n* = 23), LPP was defined as pacing proportion ≤ 71% (*n* = 7). Detailed baseline characteristics of patients were summarized in Table 1. Characteristics of patients such as gender, age, systolic blood pressure, heart rate, hypertension, diabetes, coronary heart disease, percutaneous coronary intervention (PCI) history, smoking, hemoglobin, albumin, creatinine, NT-proBNP, native QRSd, LVEF, HBP were comparable between HPP group and LPP group (all *p* > 0.05). The dosages of β-blockers in HPP group were significantly lower than those in LPP group (*p* = 0.018).

**Table 1:** Baseline clinical characteristics of patients under high and low pacing proportion.

**Variables**	**High pacing proportion**	**Low pacing proportion**	***P*-value**
N	23	7	
Gender male	15 (65.2%)	5 (71.4%)	0.760
Age (years)	74.0 ± 10.8	69.0 ± 6.6	0.157
Systolic blood pressure (mmHg)	141.7 ± 26.5	131.3 ± 26.9	0.373
Heart rate (bpm)	84.5 ± 25.2	89.4 ± 36.3	0.685
Hypertension	14 (60.9%)	5 (71.4%)	0.612
Diabetes mellitus	9 (39.1%)	3 (42.9%)	0.860
Coronary heart disease	11 (47.8%)	3 (42.9%)	0.818
PCI history	2 (8.7%)	1 (14.3%)	0.666
Smoking	9 (39.1%)	4 (57.1%)	0.400
Hemoglobin (g/L)	134.3 ± 22.6	146.7 ± 24.8	0.222
Albumin (g/L)	36.9 ± 4.4	35.7 ± 3.6	0.531
Creatinine (umol/L)	91.4 ± 22.1	92.6 ± 20.1	0.897
NT-proBNP (ng/L)	1740 (1108–4123)	3013 (1406–4908)	0.207
Native QRSd (ms)	120.0 ± 30.2	125.7 ± 29.1	0.664
LVEF (%)	46.4 ± 14.6	42.3 ± 16.3	0.525
HBP	13 (56.5%)	4 (57.1%)	0.977
Pacing proportion (%)	93.7 ± 8.6	54.1 ± 16.8	<0.001
β-blockers (mg daily)	59.9 ± 52.1	120.6 ± 68.5	0.018
Angiotensin II receptor blockers	15 (65.2%)	7 (100.0%)	0.068
Diuretics	7 (30.4%)	4 (57.1%)	0.199

*PCI, percutaneous coronary intervention; NT-proBNP, N-terminal pro-brain natriuretic peptide; QRSd, QRS duration; LVEF, left ventricular ejection fraction; HBP, His bundle pacing*.

*Data are presented as numbers (%), mean ± standard deviation, or median and interquartile range as appropriate and groups were compared using the Student's T test, Mann–Whitney U test or chi-square test as appropriate*.

### Clinical Outcomes and Echocardiographic Changes of Patients Under High and Low Pacing Proportion

After half year of HPSP, there were significant overall improvements in NYHA class, NT-proBNP and LVEF in HPP group at half-year follow-up from the baseline. There were no significant changes of NT-proBNP, LVEF and LVEDD in LPP group at half-year follow-up from the baseline (Table 2).

**Table 2:** Clinical outcomes and echocardiographic changes of patients under high and low pacing proportion.

		**NYHA class**	**NT-proBNP (ng/L)**	**LVEDD (mm)**	**LVEF (%)**
High pacing proportion	Baseline	3.1 ± 0.8	2,916 ± 2,849	55.1 ± 8.3	46.0 ± 14.7
	Half year follow-up	1.6 ± 0.7	1,187 ± 1,609	52.4 ± 7.1	53.4 ± 10.5
	*P*-value	<0.001	0.014	0.009	0.001
Low pacing proportion	Baseline	3.4 ± 0.5	3,720 ± 2,913	63.0 ± 13.7	44.1 ± 16.0
	Half year follow-up	2.5 ± 1.1	2,428 ± 2,035	62.1 ± 12.9	44.9 ± 13.7
	*P*-value	0.021	0.206	0.429	0.700

*NYHA class, New York Heart Association class; NT-proBNP, N-terminal pro-brain natriuretic peptide; LVEDD, left ventricular end diastolic dimension; LVEF, left ventricular ejection fraction*.

*Data are presented as mean ± standard deviation and groups were compared using the paired T tests*.

### Subgroup Analysis of Different QRSd and LVEF Patients for Clinical Outcomes and Echocardiographic Changes

All patients were divided into two subgroups based on QRSd: wide QRS complex group with QRS>120 ms (*n* = 15) and narrow QRS complex group with QRS ≤ 120 ms (*n* = 15); and they were also divided into two subgroups based on LVEF: the HFpEF group with LVEF ≥40% (*n* = 18) and HFrEF group with LVEF <40% (*n* = 12).

In condition of HPP (>71%), NT-proBNP was reduced to 1,085 ± 2,074 ng/L after half year of HPSP from the baseline 2,757 ± 2,835 ng/L in patients of QRS>120 ms (*p* = 0.010), and to 1,219 ± 1,032 ng/L from baseline 2,930 ± 2,897 ng/L in the patients of QRS ≤ 120 ms (*p* = 0.032). NYHA class was improved to 1.6 ± 0.9 after half year of HPSP from the baseline 3.2 ± 0.8 in patients of QRS>120 ms (*p* < 0.001), and to 1.6 ± 0.5 after HPSP from the baseline 3.0 ± 0.7 in patients of QRS ≤ 120 ms (*p* < 0.001). NT-proBNP was reduced to 1,744 ± 2,472 ng/L after half year of HPSP from the baseline 4,205 ± 4,044 ng/L in HFrEF patients (*p* = 0.032), and to 840 ± 747 ng/L from baseline 2,123 ± 1,598 ng/L in the HFpEF patients (*p* = 0.010). NYHA class was improved to 1.9 ± 1.0 after half year of HPSP from the baseline 3.5 ± 0.8 in HFrEF patients (*p* = 0.003) and to 1.4 ± 0.5 after HPSP from the baseline 2.8 ± 0.6 in HFpEF patients (*p* < 0.001).

In condition of LPP ( ≤ 71%), after half year of HPSP, there were no significant changes of NT-proBNP and NYHA class in patients of QRS>120 ms (2.259 ± 2,107 ng/L, vs. baseline 2,760 ± 1634 ng/L, *p* = 0.529; 2.8 ± 1.1, vs. baseline 3.6 ± 0.5, *p* = 0.099), and QRS ≤ 120 ms (2,709 ± 2,331 ng/L, vs. baseline 5,320 ± 4,267 ng/L, *p* = 0.359; 2.0 ± 0.0, vs. baseline 3.0 ± 0.0, *p* = 0.225). After half year of HPSP, there were no significant changes of NT-proBNP and NYHA class in HFrEF patients (2,870 ± 2,107 ng/L, vs. baseline 5,111 ± 3,399 ng/L, *p* = 0.306; 3.0 ± 0.8, vs. baseline 3.8 ± 0.5, *p* = 0.058)and HFpEF patients (1,985 ± 2,174 ng/L, vs. baseline 2,329 ± 1,756 ng/L, *p* = 0.363; 2.0 ± 1.2, vs. baseline 3.0 ± 0.0, *p* = 0.182).

**Figure 5:**
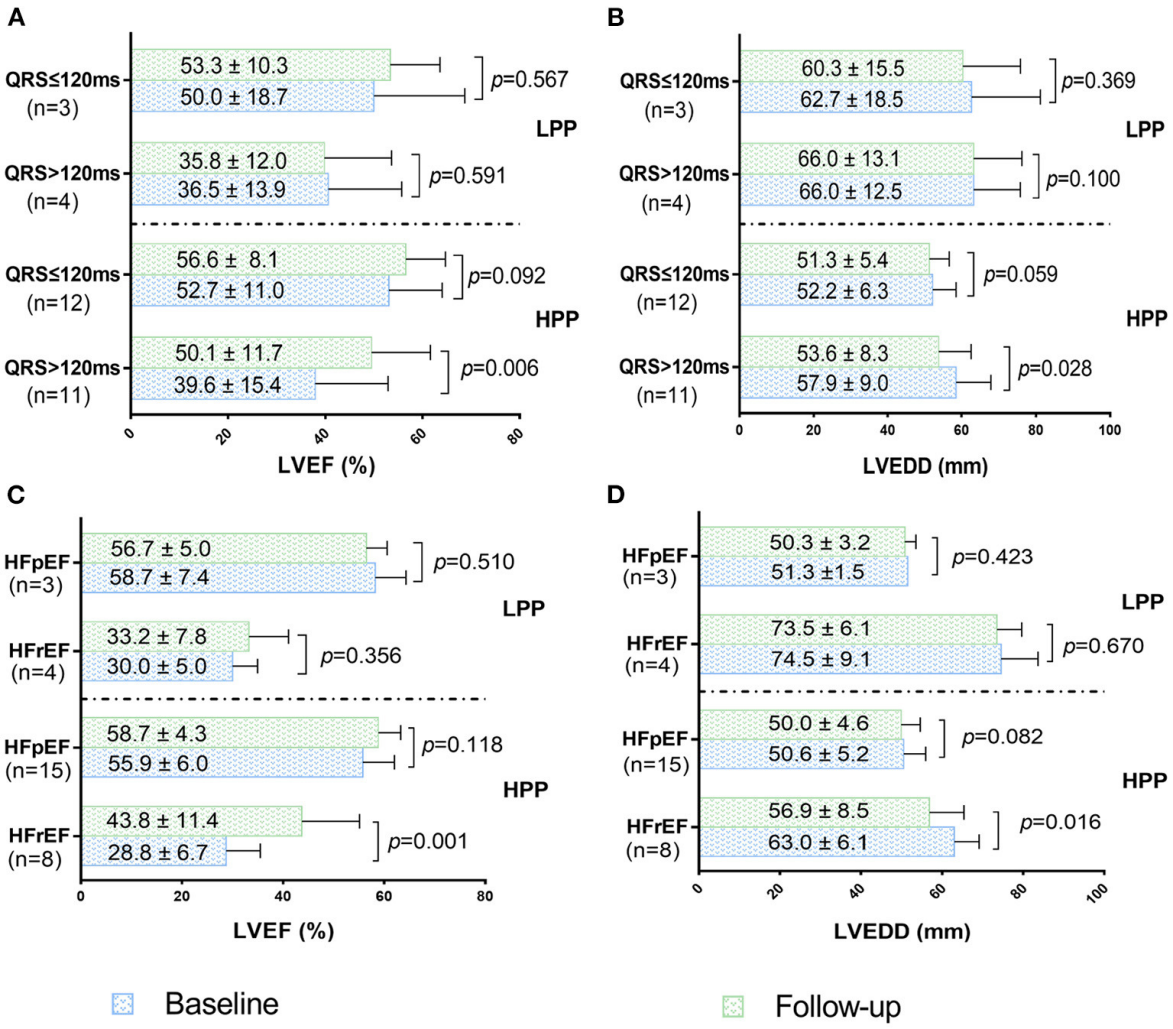
LVEF changes of patients with wide QRS complex and narrow QRS complex under HPP and LPP **(A)**. LVEDD changes of patients with wide QRS complex and narrow QRS complex under HPP and LPP **(B)**. LVEF changes of patients with HFpEF and HFrEF under HPP and LPP **(C)**. LVEDD changes of patients with HFpEF and HFrEF under HPP and LPP **(D)**. HPP, high pacing proportion; LPP, low pacing proportion; LVEF, left ventricular ejection fraction; LVEDD, left ventricular end diastolic dimension; HFpEF, heart failure with preserved ejection fraction; HFrEF, heart failure with reduced ejection fraction.

Echocardiographic changes of HPP and LPP patients with different QRSd and LVEF were summarized in Figure 5. Compared with baseline echocardiographic parameters, LVEF significantly increased while LVEDD decreased in HFrEF and wide QRS complex (QRS>120 ms) patients when pacing proportion>71%. However, moderate but no significant improvements of LVEF and LVEDD were observed in HFpEF and narrow QRS complex (QRS ≤ 120 ms) patients. In condition of pacing proportion ≤ 71%, HFrEF and HFpEF patients showed no significant change in LVEF and LVEDD after half year of HPSP treatment regardless of QRSd (Figure 5).

### Kaplan-Meier Curves Analysis for MACE Under High and Low Proportion

**Figure 6:**
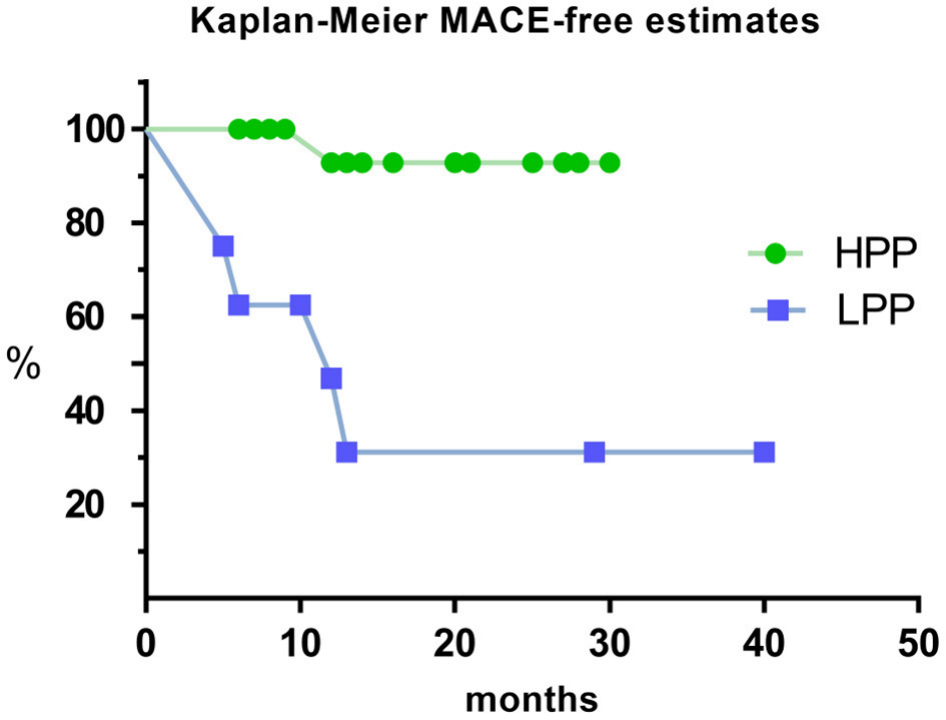
Kaplan–Meier estimate for MACE of HPP and LPP. MACE: composite endpoint of readmission for HF and cardiac mortality. MACE, major adverse cardiovascular events; HPP, high pacing proportion; LPP, low pacing proportion.

Kaplan-Meier survival curves analysis were performed for MACE in all patients under different pacing proportion. It showed that patients in LPP had significantly higher MACE rate than patients in HPP (Log Rank test, *p* < 0.001; Figure 6).

## Discussion

Ventricular rhythm is irregular in AF with or without rapid ventricular rate. Fast ventricular rate has an acknowledged deleterious impact upon left ventricular systolic function (14, 15), and ventricular irregular rhythm itself also has adverse effects on left ventricular systolic function (4). Therefore, the treatment of AF should focus on rate control and rhythm control. HPSP combined with AVN ablation can not only achieve rate control and rhythm control, but also utilize the complete His-Purkinje pathway, which is beneficial to synchronous ventricular activation (16). If AF is complicated with BBB and wide QRS complex, whether LBBB or RBBB pattern, HPSP can overcome ventricular systolic asynchrony and improve cardiac function by correcting conduction block (17, 18). In patients with AF without AVN ablation,β-blockers can inhibit AVN conduction function and reduce intrinsic heart rate to achieve high proportion of HPSP and fulfill the purpose of rate control and rhythm control.

In this study, we found that higher pacing proportion of HPSP could significantly improve the clinical outcomes and echocardiographic results of AF patients complicated with HF. The clinical characteristics such as age, gender, co-morbidities, hepatic and renal function, cardiac function in HPP patients were similar to those in LPP. Therefore, the discrepancy of therapeutic effect between HPP and LPP could be attributed to pacing proportion itself. Nabeta et al. demonstrated that increasing the dose of β-blockers was an independent factor to improve the prognosis of HF patients treated with CRT (19). In our study, β-blockers dosage (59.9 ± 52.1 mg daily) (medication duration 5.04 ± 1.19 months) in HPP was significantly lower than that (120.6 ± 68.5 mg daily) (medication duration 4.71 ± 1.25 months) in LPP (*p* = 0.018), indicating that the clinical benefits of patients in HPP were further ascribed to the higher pacing proportion.

Boczar et al. showed that improvements in HF symptoms using NYHA classification based on severity, reduction of LVEDD, improvement of LVEF, were observed CRT in wide QRS complex (159.2 ± 28.6 ms) patients with AF and HF by implanting HB lead (17). In this study, HBP achieved an average pacing percentage of 97% through the optimization of medical therapy and appropriate device programming (17). Hayes et al. observed the proportion of biventricular pacing (BVP) > 98% could significantly reduce mortality rate (20). Jacobsson et al. also demonstrated that AF was associated with poor prognosis in patients with CRT, due to AF resulting in a decrease in the proportion of BVP. The proportion of BVP ≤ 98% was an independent risk factor for poor prognosis in patients with CRT (21). Our study found that the effect could be observed when the proportion of HPSP was more than 71% in wide QRS complex AF patients with HF. The relatively low pacing ratio of HPSP to achieve therapeutic effect seemly presented its potential advantages over BVP. Furthermore, Arnold et al. demonstrated BVP still produced a non-physiological activation pattern (22), indicating the physiology of BVP inferior to that of HPSP, and Arnold et al. also found that HBP could provide better ventricular resynchronization and hemodynamic feedback than BVP (22). The study above gave us the plausibility of HPSP superior to BVP for HF complicated with AF. However, in 2019, a pilot head-to-head study comparing HBP with BVP demonstrated that HBP produced greater QRSd reduction than BVP, but no significant improvements in echocardiographic parameters as compared with BVP (23). In this first randomized controlled trials (RCT), high rate of crossover from HBP to BVP compromised the assessment of HBP efficacy, and half crossover exhibited non-specific intraventricular conduction delay which cannot be corrected by HBP, thus efficacy of HBP was offset. The discrepancy of pacing proportion in our study might be caused by not only pacing modes, but also the difference of native QRSd in study population. QRSd of the wide QRS complex patients in our study was 146.1 ± 18.9 ms, thus the degree of ventricular activation asynchrony was lower than that of the patients with CRT implantation (17, 21). The detrimental impact of native activation on ventricular remodeling was relatively low, suggesting a relatively low pacing proportion needed to achieve clinical benefits. On the basis of the above reasons, it is preliminarily explained the relatively low pacing proportion sufficient to improve the clinical condition compared with previous studies. Previous studies on paroxysmal AF complicated with HF revealed that the longer the sinus rhythm (SR) time (≥61%) was maintained, the more significant the improvement of life quality, 6-min walk test and NYHA classification were observed (24). For persistent AF patients with HPSP, ventricular activation sequence and rhythm are similar to those of SR. However, considering AF has not been corrected, the atrium loses contraction function and impairs 20% of cardiac output compared with SR in patients with paroxysmal AF (25). Therefore, SR time >61% was enough to achieve the purpose of treatment. Furthermore, QRSd of the patients included in this study was 114 ± 30 ms (24), ventricular synchronization was even better than that of our study (QRSd = 121.4 ± 29.5 ms). As for HPSP application for persistent AF patients with HF, the analysis above gives a tendency that when the native QRSd is greater than pacing QRSd, the longer the native QRSd is, the higher pacing proportion of resynchronization therapy is needed to achieve clinical benefits. There could be a lower limit for the pacing proportion required for the effective treatment, but the establishment of the lower limit still needs further exploration.

In narrow QRS complex (<120 ms) persistent AF patients with HF, regular paced ventricular rhythm by HPSP was a primary hemodynamic benefit due to the absence of BBB (26). Our study found that there were significant improvement of NYHA class and NT-proBNP when HPP was applied, but no significant improvements were observed in echocardiographic measurements. Deshmukh et al. performed AVN ablation and HBP in patients with narrow QRS complex (<120 ms) AF and dilated cardiomyopathy, which showed the improvement of LVEDD and LVEF (11). Huang et al. implemented AVN ablation and HBP for patients with narrow QRS complex (107.1 ± 25.8 ms) AF and HF, and the echocardiographic parameters were also improved (12). Compared with the results of previous studies, the difference in the improvement of echocardiography in our study was related to the fact that our subjects did not undergo AVN ablation, and the pacing proportion was <100%. Therefore, AVN ablation was recommended for these patients to increase the pacing proportion to 100%, in order to further improve the therapeutic effect.

Our study found that HPSP proportion had a good predictive ability for MACE in persistent AF patients with HF (AUC = 0.830). The optimal cut-off point of pacing proportion related to prognosis was 71% during the follow-up period of 15.1 ± 9.4 months. Patients with QRSd > 120 ms or HFrEF in HPP group could showed significant improvement in clinical outcomes and echocardiographic results within 6 months after HPSP, which were similar to the results of Huang et al. (12). However, unlike previous studies (12), patients with QRSd ≤ 120 ms or HFpEF in HPP group showed modest, but no significant improvement in echocardiographic results. The discrepancy perhaps resulted from not only the absence of AVN ablation and pacing proportion being <100%, but also follow-up time of 6 months significantly shorter than follow-up time of 21.1 ± 9.3 months of Huang et al. (12). Although there is no clinical evidence of HPSP superior to BVP for patients with HF, 23 patients (76.7%) with LVEF>35% or QRS <130 ms in our study are not candidates for BVP according to 2021 ESC guidelines (9), and results of our study indicate the potential of HPSP in patients with HF who are not eligible for BVP, the potential as an alternative strategy to CRT (27), and the promising future for AF patients with HF.

Our study has some limitations that should be mentioned. This was a retrospective, observational single center study with a small patient population. We expected to perform a large-scale multicenter prospective clinical trial in the future. Furthermore, this study belonged to the self-control study and lacked a control group, so the differences in therapeutic effects of the HPSP group, internal medicine treatment group and catheter ablation group could not be obtained. Randomized controlled trials are expected to be conducted in the future to compare the differences in therapeutic effects of each treatment method. In this study, patients with pacing proportion >71% achieved significant clinical benefits. However, given the limited size of the study population and unevenly distributed pacing proportion, the pacing proportion amounting to 71% could only indicate that the higher the pacing proportion, the greater the clinical benefit. And it could not be interpreted as the lower limit of pacing proportion to achieve effective therapeutic effect. Large-scale observations are necessary to establish a lower limit for pacing proportion.

## Data Availability Statement

The original contributions presented in the study are included in the article/supplementary material, further inquiries can be directed to the corresponding author.

## Ethics Statement

Written informed consent was obtained from the individual(s) for the publication of any potentially identifiable images or data included in this article.

## Author Contributions

All authors listed have made a substantial, direct, and intellectual contribution to the work and approved it for publication.

## Conflict of Interest

The authors declare that the research was conducted in the absence of any commercial or financial relationships that could be construed as a potential conflict of interest.

## Publisher's Note

All claims expressed in this article are solely those of the authors and do not necessarily represent those of their affiliated organizations, or those of the publisher, the editors and the reviewers. Any product that may be evaluated in this article, or claim that may be made by its manufacturer, is not guaranteed or endorsed by the publisher.
